# The Association Between Anti-Thyroid Peroxidase Antibody and Dyslipidemia in Subclinical Hypothyroidism Among the Rural Population of Central India

**DOI:** 10.7759/cureus.22317

**Published:** 2022-02-17

**Authors:** Manoj Kumar, Dheeraj Dheeraj, Ravi Kant, Ashok Kumar

**Affiliations:** 1 Medicine, Uttar Pradesh University of Medical Sciences, Saifai, IND; 2 General Medicine, All India Institute of Medical Sciences, Rishikesh, Rishikesh, IND

**Keywords:** lipid profiles, thyroid peroxidase antibodies, cardiovascular prevention, dyslipidemia, thyroid disorder, subclinical hypothryroidism

## Abstract

Objective

In this study, we aimed to determine the association between anti-thyroid peroxidase (anti-TPO) antibody and dyslipidemia in subclinical hypothyroidism (SCH).

Materials and methods

We conducted a cross-sectional case-control study in the department of medicine at a tertiary care teaching hospital in central India. The study consisted of 150 patients (75 cases and 75 controls) who fulfilled the inclusion and exclusion criteria.

Results

The study showed that serum cholesterol was high in 23.1% of cases in the negative anti-TPO antibody SCH sub-group and 88.7% of cases in the positive anti-TPO antibody SCH sub-group (p<0.001). Serum triglyceride (TG) levels were high in 61.5% of cases in the negative anti-TPO antibody SCH sub-group and 90.3% of cases in the positive anti-TPO antibody SCH sub-group (p=0.008). Serum high-density lipoproteins (HDL) were low in 15.4% of cases in the negative anti-TPO antibody SCH sub-group and 24.2% of cases in the positive anti-TPO antibody SCH sub-group (p=0.490). Serum low-density lipoproteins (LDL) were high in 61.5% of cases in the negative anti-TPO antibody SCH sub-group and 90.3% of cases in the positive anti-TPO antibody SCH sub-group (p=0.008). Serum very-low-density lipoproteins (VLDL) were high in 84.6% of cases in the negative anti-TPO antibody SCH sub-group and 83.9% of cases in the positive anti-TPO antibody SCH sub-group (p=0.947). Based on our findings, 82.8% of participants with negative anti-TPO antibodies had normal serum LDL levels, while 11.1% of participants with positive anti-TPO antibodies had normal LDL levels (p<0.001). The study also showed that elevated serum LDL was present in 17.2% of participants with negative anti-TPO antibody levels and 88.9% of participants with positive anti-TPO antibodies (p<0.001). The study showed a correlation coefficient of 0.0432 between serum TG and anti-TPO antibody levels (p<0.001).

Conclusion

Our findings showed an increased incidence of dyslipidemia in SCH patients with positive anti-TPO antibodies. SCH with positive anti-TPO antibody is significantly associated with elevated serum total cholesterol (TC) levels, serum TG levels, and serum LDL levels. Hence, early screening and diagnosis of dyslipidemia are crucial to prevent cardiovascular morbidity and mortality in SCH patients with positive anti-TPO antibodies.

## Introduction

The American Thyroid Association defines subclinical hypothyroidism (SCH) as a condition with elevated thyroid-stimulating hormone (TSH) levels accompanied by thyroid hormone levels in the normal range. However, this definition is only applicable when thyroid function has been stable, the hypothalamic-pituitary-thyroid axis is normal, and in the absence of any recent or ongoing severe illness [1]. The importance of studying SCH lies in the fact that it is much more common than overt hypothyroidism. The National Health and Nutrition Examination Survey (NHANES III) found the prevalence of subclinical disease to be 4.3%, while that of overt disease was 0.3%. The Colorado Thyroid Disease Prevalence Study has reported a prevalence of 8.5% for subclinical disease and 0.4% for overt disease [1].

Lipid profile changes in overt hypothyroidism are well established; however, the results regarding lipid profile changes in SCH have been controversial. Althaus et al., in their study of SCH, found a 10% increase of serum low-density lipoprotein (LDL) (p<0.01) and a 10% decrease of high-density lipoprotein (HDL) (p<0.05) while there were no significant changes in total cholesterol (TC) and serum triglyceride (TG) levels [2]. However, Hueston et al. concluded that SCH does not appear to be associated with abnormalities in serum cholesterol or TG levels [3]. Additionally, there have been very few Indian studies on this condition, especially among SCH patients with positive anti-thyroid peroxidase (TPO) antibodies. Srivastava et al. studied the association between TPO antibodies and dyslipidemia among 50 adult SCH patients and concluded that in SCH patients, dyslipidemia is significantly associated with anti-TPO antibody positivity, especially in females [4].

As mentioned above, there is scant regional data on the relationship between anti-TPO antibodies and dyslipidemia in SCH. For early screening and diagnosis of patients at risk of cardiovascular events, it is crucial to study the association between TPO antibodies and dyslipidemia in SCH. In this study, we aimed to analyze the association between dyslipidemia and anti-TPO antibodies in patients with SCH.

## Materials and methods

Study design and setting

This was a cross-sectional case-control study conducted in the outpatient section of the department of internal medicine at a tertiary care teaching hospital in a rural region of central India.

Patient selection

**Figure 1:**
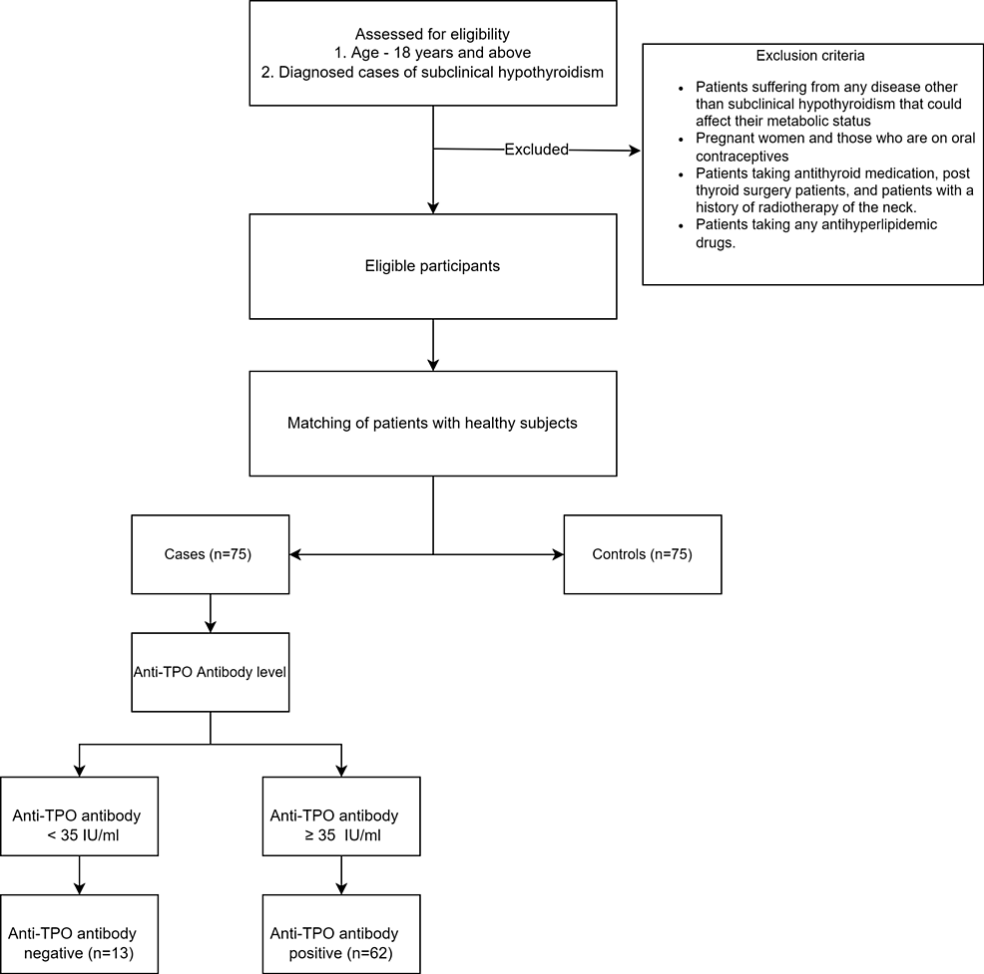
Study flow chart TPO: thyroid peroxidase

The inclusion criteria were patients aged 18 years and above with a diagnosed case of SCH as per the American Thyroid Association guidelines [1]. Diagnosed cases of SCH were further subdivided into those with negative anti-TPO levels (anti-TPO level <35 IU/ml) and positive anti-TPO levels (anti-TPO level ≥35 IU/ml). We also included age, sex, and race-matched healthy subjects as controls. Patients suffering from any disease other than SCH that could affect their metabolic status, such as end-stage renal disease, chronic liver disease, post-myocardial infarction, patients with congestive heart failure, and those with diabetes were excluded. Pregnant women and those who were on oral contraceptives were also excluded. We also excluded patients on antithyroid medication, post-thyroid surgery patients, and those with a history of radiotherapy of the neck. Patients taking any antihyperlipidemic drugs were also excluded. The study flow chart is illustrated in Figure 1.

Methods

A serum sample was collected in a fasting state (12-hour fasting state) with a sterile venipuncture technique. The sample was then processed for lipid profile, thyroid profile (TSH, free T3, and free T4), and anti-TPO antibody. Lipid estimations were done using an automated analyzer and commercial kits. We adhered to the National Cholesterol Education Program Adult Treatment Panel III guidelines for serum lipids. According to these guidelines, hypercholesterolemia is defined as a TC value ≥200 mg/dl, high LDL as an LDL level ≥130 mg/dl, hypertriglyceridemia as a TG value ≥150 mg/dl, and low high-density lipoprotein (HDL) as an HDL value <40 mg/dl [5]. Anti-TPO antibody was analyzed using 1000SR (Abbott Laboratories, Chicago, IL) with the normal value being <35 IU/ml. Participants with a value ≥35 IU/ml were considered anti-TPO antibody-positive.

Study sample size

The sample size of this case-control study comprised a total of 150 individuals. A total of 75 patients diagnosed with SCH were included in the case group and 75 healthy matched individuals were recruited as the control group.

Data entry and analysis

Statistical analysis was performed using SPSS Statistics version 23 (IBM, Armonk, NY). Continuous variables were presented as mean ± standard deviation (SD) and categorical variables were presented as absolute numbers and percentages. Differences between groups were assessed with the chi-squared or Fisher's exact test for categorical variables as appropriate. The association of anti-TPO antibodies with various components of lipid level was assessed using multiple regressions. A p-value <0.05 was considered statistically significant.

Ethical considerations

The study was conducted after obtaining approval from the Ethics Committee of Uttar Pradesh University of Medical Sciences.

Confidentiality of data

Strict confidentiality was maintained regarding the information obtained about the patients, and the identity of the patients was not revealed. Data, if shared, were anonymized.

## Results

The study consisted of 150 patients (75 cases and 75 controls) satisfying the inclusion and exclusion criteria; 77.3% of the subjects were female and 22.7% were male. The study showed anti-TPO positivity among 82.7% of cases of SCH as depicted in Table 1. Dyslipidemia was present in 36% among the control group and 100% in the case group (p<0.001).

**Table 1:** Anti-TPO antibody distribution among SCH cases TPO: thyroid peroxidase; SCH: subclinical hypothyroidism

Anti-TPO antibody	Number of cases (%)
Positive anti-TPO antibody (≥35 IU/ml)	62 (82.7%)
Negative anti-TPO antibody (<35 IU/ml)	13 (17.3%)
Total	75

This study also demonstrated the association between lipid profile and anti-TPO antibody as depicted in Table 2. Among SCH patients, the positive anti-TPO antibody sub-group had hypercholesteremia in 88.7% of cases, whereas in the negative anti-TPO antibody sub-group, hypercholesteremia was present in 23.1% of cases (p<0.001). Likewise, in SCH patients, hypertriglyceridemia was found in 90.3% of cases of positive anti-TPO antibody sub-group, while in the negative anti-TPO antibody sub-group, hypertriglyceridemia was present in 61.5% of cases (p=0.008). In the positive anti-TPO antibody sub-group, 90.3% of cases had raised LDL levels, while in the negative anti-TPO antibody sub-group, raised LDL levels were present in 61.5% of cases (p=0.008).

**Table 2:** Correlation between abnormal lipid and anti-TPO antibody in SCH cases SCH: subclinical hypothyroidism; TPO: thyroid peroxidase; TC: total cholesterol; TG: triglyceride; HDL: high-density lipoprotein; LDL: low-density lipoprotein; VLDL: very-low-density lipoprotein

Parameters	TPO		P-value
	Negative (<35 IU/ml), n (%)	Positive (≥35 IU/ml), n (%)	
TC ≥200	3 (23.1%)	55 (88.7%)	<0.001
Serum TG ≥150	8 (61.5%)	56 (90.3%)	0.008
HDL <40	2 (15.4%)	15 (24.2%)	0.490
LDL ≥130	8 (61.5%)	56 (90.3%)	0.008
VLDL ≥30	11 (84.6%)	52 (83.9%)	0.947

Our study showed that serum HDL was low in 15.4% of cases in the negative anti-TPO antibody SCH sub-group and 24.2% of cases in the positive anti-TPO antibody SCH sub-group (p=0.490). Serum VLDL was elevated in 84.6% of cases in the negative anti-TPO antibody SCH sub-group and 83.9% of cases in the positive anti-TPO antibody SCH sub-group (p=0.947). Additionally, 82.8% of participants with anti-TPO antibodies within the normal range had normal LDL levels, while 11.1% of participants with elevated anti-TPO antibodies had normal LDL levels (p<0.001) as depicted in Table 3. Also, elevated LDL was present in 17.2% of participants with normal anti-TPO antibody levels and 88.9% of participants with elevated anti-TPO antibodies (p<0.001) as depicted in Table 3.

**Table 3:** Correlation between anti-TPO antibodies and elevated LDL among all participants TPO: thyroid peroxidase; LDL: low-density lipoproteins

Anti-TPO antibody	LDL <100, n (%)	LDL ≥100, n (%)	P-value
Negative anti-TPO antibody (<35 IU/ml)	72 (82.8%)	15 (17.2%)	<0.0001
Positive anti-TPO antibody (≥35 IU/ml)	7 (11.1%)	56 (88.9%)	

**Figure 2:**
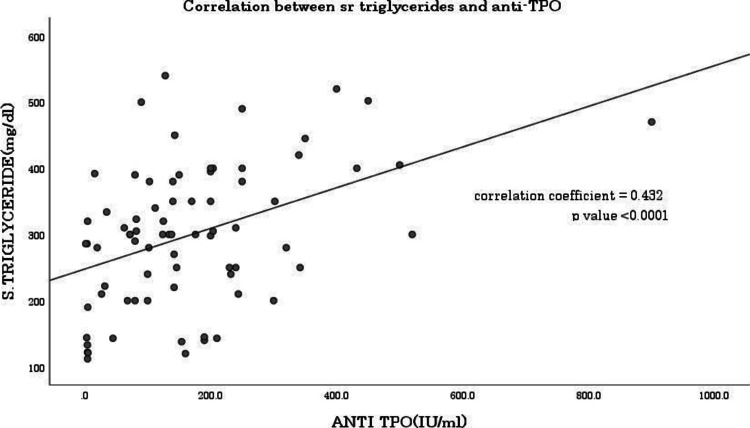
Correlation between anti-TPO antibody and triglyceride TPO: thyroid peroxidase

The study showed a correlation coefficient of 0.0432 between serum TG and anti-TPO antibody level as shown in Figure 2.

## Discussion

SCH is defined as a state of elevated TSH level with a normal level of thyroid hormones. Various epidemiological studies in India have shown a prevalence rate of 9-11.4% for SCH among the general population [6]. SCH is associated with an increased risk of coronary artery disease, cerebrovascular disease, and heart failure, and the risk increases with an increasing level of TSH [7]. Studies have shown a strong association between dyslipidemia and SCH [8]. We conducted a prospective study to examine the association between anti-TPO antibody and dyslipidemia in SCH among the rural population of central India.

In their study of 50 cases, Jayashankar et al. found anti-TPO positivity among 50% of SCH cases [9], whereas Mohanty et al. showed that 73% of SCH cases had positive anti-TPO levels [10]. The current study found anti-TPO positivity among 82.7% of cases of SCH. Hussain et al., in their study on the effects of dyslipidemia on SCH, showed a significant negative correlation between FT4 and serum cholesterol and a nonsignificant correlation between TSH and serum TG, serum HDL, as well as serum LDL [8]. Similarly, Ejaz et al. showed an increased incidence of dyslipidemia and significantly elevated levels of serum cholesterol and serum LDL in patients with SCH [11]. The current study showed a significant association between anti-TPO positivity and raised serum cholesterol, raised serum TG levels, and raised serum LDL levels; however, a nonsignificant association was found between anti-TPO positivity and serum HDL and serum VLDL.

Srivastava et al. in their study found that dyslipidemia was present in 100% of SCH patients with positive anti-TPO antibodies [4]. In the current study, dyslipidemia was present in 36% of subjects in the control group and 100% of subjects in the case group. A significant difference was found in dyslipidemia proportion between cases and controls (p<0.001). We found a correlation coefficient of 0.432 (p<0.001) between anti-TPO antibody and serum TG as depicted in Figure 2, indicating a positive correlation between the two variables, i.e., when the value of one variable increases, the value of the other variable also tends to increase.

**Figure 3:**
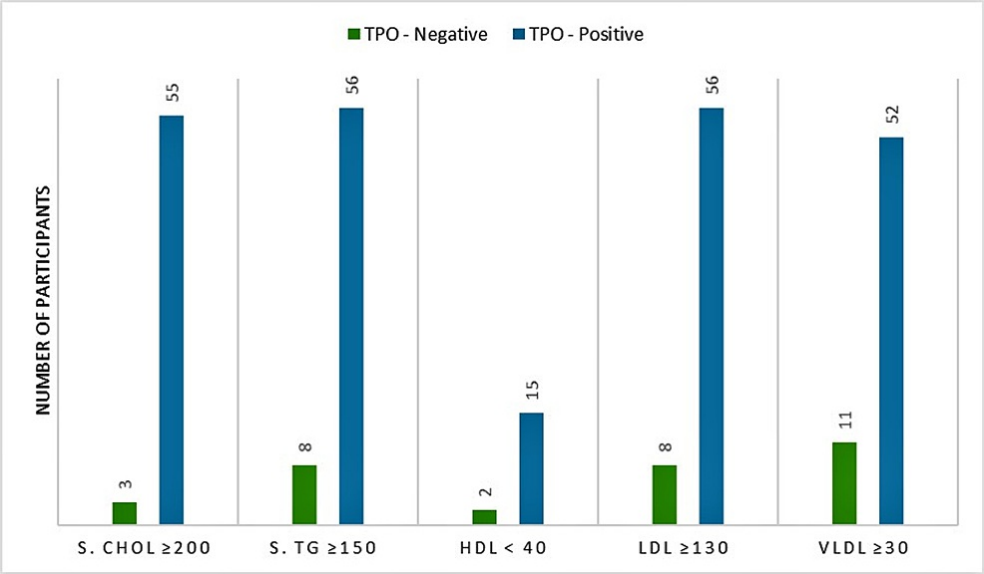
Correlation between lipid profile and anti-TPO antibodies TPO: thyroid peroxidase; S. Chol: serum cholesterol; S.TG: serum triglyceride: HDL: high-density lipoprotein; LDL: low-density lipoprotein; VLDL: very-low-density lipoprotein

The current study, while exploring the association of lipid profile with anti-TPO antibody, found that SCH with anti-TPO antibody positivity is significantly associated with raised serum cholesterol as well as raised serum TG and serum LDL levels as depicted in Figure 3. Asranna et al. have shown a TSH-dependent increase in cholesterol, LDL, VLDL, and TG levels in their study and concluded that achieving euthyroid status has a favorable effect on lipid profile [12].

In the Rotterdam study, SCH was present in 10.8% of participants and was associated with a greater age-adjusted prevalence of aortic atherosclerosis (odds ratio: 1.7%, 95% CI: 1.1-2.6) and myocardial infarction (odds ratio: 2.3, 95% CI: 1.3-4.0) [13]. It was shown that the incidence of atherosclerosis was even higher in SCH if the anti-TPO antibody was positive. Dey et al. have concluded that diagnosis and treatment of SCH will have possible cardioprotective benefits [14].

To summarize the findings, our study has found a high prevalence of anti-TPO positivity in SCH. Additionally, we have found a significant association of dyslipidemia with anti-TPO positivity. The association between dyslipidemia and SCH is of great significance as dyslipidemia is a well-known risk factor for hypertension, cardiovascular disease, and stroke. The current study further highlights the importance of routine monitoring of lipid profiles in cases of SCH to timely identify any derangements to prevent adverse outcomes. We strongly recommend further investigation on the use of thyroxine in patients with SCH for dyslipidemia.

To the best of our knowledge, this is the first study conducted on this topic in a rural region of central India; however as this study was conducted at a single institute, further studies are required to validate our findings before we can extrapolate them to the general population. Secondly, as the nature of our study design was case-control, this study can be used to establish a correlation but cannot establish causation. Hence, a large-scale prospective study is required to confirm the association between dyslipidemia and SCH.

## Conclusions

This study showed an increased incidence of dyslipidemia in patients with elevated anti-TPO antibodies. SCH with positive anti-TPO antibody is significantly associated with elevated serum TC, serum TG, and serum LDL levels. Dyslipidemia is a significant risk factor for cardiovascular disease and mortality. Hence, early screening and timely diagnosis of dyslipidemia are recommended to prevent cardiovascular morbidity and mortality in SCH patients with positive anti-TPO antibodies. Further, we recommend exploring the role of thyroxine as an intervention to target the problem of dyslipidemia in patients with SCH, particularly in anti-TPO antibody-positive patients.

## References

[1] Garber JR, Cobin RH, Gharib H (2012). Clinical practice guidelines for hypothyroidism in adults: cosponsored by the American Association of Clinical Endocrinologists and the American Thyroid Association. Thyroid.

[2] Althaus BU, Staub JJ, Ryff-De Lèche A, Oberhänsli A, Stähelin HB (1988). LDL/HDL-changes in subclinical hypothyroidism: possible risk factors for coronary heart disease. Clin Endocrinol (Oxf).

[3] Hueston WJ, Pearson WS (2004). Subclinical hypothyroidism and the risk of hypercholesterolemia. Ann Fam Med.

[4] Srivastava VK, Singh H (2017). Association of thyroid peroxidase antibody and dyslipidemia in subclinical hypothyroidism. J Family Med Prim Care.

[5] Fedder DO, Koro CE, L'Italien GJ (2002). New National Cholesterol Education Program III guidelines for primary prevention lipid-lowering drug therapy: projected impact on the size, sex, and age distribution of the treatment-eligible population. Circulation.

[6] Deshmukh V, Behl A, Iyer V, Joshi H, Dholye JP, Varthakavi PK (2013). Prevalence, clinical and biochemical profile of subclinical hypothyroidism in normal population in Mumbai. Indian J Endocrinol Metab.

[7] Redford C, Vaidya B (2017). Subclinical hypothyroidism: should we treat?. Post Reprod Health.

[8] Hussain A, Elmahdawi AM, Elzeraidi NE, Nouh F, Algathafi K (2019). The effects of dyslipidemia in subclinical hypothyroidism. Cureus.

[9] Jayashankar CA, Avinash S, Shashidharan B, Vijaya S, Shruthi KR, Nikethan D, Harshavardhan J (2017). The prevalence of anti-thyroid peroxidase antibodies in subclinica and clinical hypothyroid patients. Int J Res Med Sci.

[10] Mohanty S, Amruthlal W, Reddy GC, Kusumanjali G, Kanagasabapathy AS, Rao P (2008). Diagnostic strategies for subclinical hypothyroidism. Indian J Clin Biochem.

[11] Ejaz M, Kumar P, Thakur M (2021). Comparison of lipid profile in patients with and without subclinical hypothyroidism. Cureus.

[12] Asranna A, Taneja RS, Kulshreshta B (2012). Dyslipidemia in subclinical hypothyroidism and the effect of thyroxine on lipid profile. Indian J Endocrinol Metab.

[13] Hak AE, Pols HA, Visser TJ, Drexhage HA, Hofman A, Witteman JC (2000). Subclinical hypothyroidism is an independent risk factor for atherosclerosis and myocardial infarction in elderly women: the Rotterdam Study. Ann Intern Med.

[14] Dey A, Kanneganti V, Das D (2019). A study of the cardiac risk factors emerging out of subclinical hypothyroidism. J Family Med Prim Care.

